# Use of diffusion-weighted MRI to modify radiosurgery planning in brain metastases may reduce local recurrence

**DOI:** 10.1007/s11060-016-2320-9

**Published:** 2016-11-14

**Authors:** Rasheed Zakaria, Andreas Pomschar, Michael D. Jenkinson, Jörg-Christian Tonn, Claus Belka, Birgit Ertl-Wagner, Maximilian Niyazi

**Affiliations:** 1 Department of Neurosurgery, The Walton Centre NHS Foundation Trust, Lower Lane, Fazakerley, Liverpool, L9 7LJ UK; 2 Institute of Integrative Biology, University of Liverpool, Liverpool, UK; 3 Institute of Clinical Radiology, LMU Munich, Munich, Germany; 4 Department of Neurosurgery, LMU Munich, Munich, Germany; 5 Department of Radiation Oncology, LMU Munich, Munich, Germany; 6 German Cancer Research Centre (DKFZ), Heidelberg, Germany; 7 German Cancer Consortium (DKTK), Heidelberg, Germany; 8 Institute of Translational Medicine, University of Liverpool, Liverpool, UK

**Keywords:** Brain metastasis, Stereotactic radiosurgery, ADC, DWI, Planning study

## Abstract

Stereotactic radiosurgery (SRS) is an effective and well tolerated treatment for selected brain metastases; however, local recurrence still occurs. We investigated the use of diffusion weighted MRI (DWI) as an adjunct for SRS treatment planning in brain metastases. Seventeen consecutive patients undergoing complete surgical resection of a solitary brain metastasis underwent image analysis retrospectively. SRS treatment plans were generated based on standard 3D post-contrast T1-weighted sequences at 1.5T and then separately using apparent diffusion coefficient (ADC) maps in a blinded fashion. Control scans immediately post operation confirmed complete tumour resection. Treatment plans were compared to one another and with volume of local recurrence at progression quantitatively and qualitatively by calculating the conformity index (CI), the overlapping volume as a proportion of the total combined volume, where 1 = identical plans and 0 = no conformation whatsoever. Gross tumour volumes (GTVs) using ADC and post-contrast T1-weighted sequences were quantitatively the same (related samples Wilcoxon signed rank test = −0.45, p = 0.653) but showed differing conformations (CI 0.53, p < 0.001). The diffusion treatment volume (DTV) obtained by combining the two target volumes was significantly greater than the treatment volume based on post contrast T1-weighted MRI alone, both quantitatively (median 13.65 vs. 9.52 cm^3^, related samples Wilcoxon signed rank test p < 0.001) and qualitatively (CI 0.74, p = 0.001). This DTV covered a greater volume of subsequent tumour recurrence than the standard plan (median 3.53 cm^3^ vs. 3.84 cm^3^, p = 0.002). ADC maps may be a useful tool in addition to the standard post-contrast T1-weighted sequence used for SRS planning.

## Introduction

Brain metastases are increasingly common and cause significant morbidity and mortality in patients with solid tumours [1–4]. Recent analyses of the brain–brain metastasis interface [5, 6] suggest local invasion may be more important than previously thought [7] and various solutions including supra-marginal surgical resection and cavity boost stereotactic radiosurgery (SRS) have been proposed [8–10]. MRI scans of the brain are routinely obtained in the course of diagnosis and disease monitoring for these patients and diffusion MRI (DWI) sequences are commonly included, historically to distinguish these ring enhancing tumours from abscesses but more recently to aid in differentiating solitary metastases from glioma, for surgical planning and even to predict prognosis [11]. It is known that in human brain metastases the apparent diffusion coefficient (ADC) increases with decreasing cellularity [12] and decreasing density of intratumoral connective tissue [13]. Changes in ADC in the peritumoral brain may precede micro-metastasis appearance in animal models [14] and lower tumour ADC values have been shown to be predictive of earlier recurrence and shorter survival after neurosurgery [12, 13] and SRS [15]. The aim of this study was to investigate whether the addition of an ADC map to a standard post-contrast T1 weighted sequence would aid SRS planning, in terms of increasing the final planned treatment volume or potentially encompassing peritumoral areas where local recurrence subsequently occurred.

## Materials and methods

### Patients

In this retrospective study, 17 consecutive neurosurgical patients (median age 54 years, 4 male/13 female) with a supratentorial brain metastasis who had undergone diffusion-weighted MRI (DWI) as part of their preoperative investigations and who had developed recurrence at the site of surgery (as per generic RANO criteria [16]) were included (Table 1 **)**. All patients studied underwent gross total resection and there were no complications within 30 days of operation. Median overall survival was 11.8 months (95% CI 7.8–15.8 months) and median time to local recurrence was 8.0 months (95% CI 6.6–9.4 months).

**Table 1:** Demographic and clinical information of the included patients

ID	Age (years)	Sex	Performance status (KPS) (%)	GPA category	Primary cancer	Extracranial metastases	Status of primary tumour	Location of brain metastasis
M011	38	F	100	3.0	Melanoma (BRAF+)	Present	Stable disease	Occipital
M013	48	F	90	3.5–4.0	NSCLC	Absent	Stable disease	Frontal
M019	70	F	90	1.5–2.5	Lung NOS	Present	Synchronous	Cerebellar
M028	57	F	70	1.5–2.5	Melanoma	Present	Synchronous	Parietal
M031	70	M	90	3.0	NSCLC	Absent	Stable disease	Occipital
M033	36	F	100	3.5–4.0	NSCLC	Absent	Synchronous	Temporal
M042	60	F	70	1.5–2.5	Unknown	Present	Synchronous	Frontal
M052	48	F	90	3.0	Breast (HER2−)	Present	Stable disease	Parietal
M222	56	F	90	3.5–4.0	NSCLC	Absent	Stable disease	Frontal
M302	35	F	90	3.0	Melanoma (BRAF+)	Present	Stable disease	Parietal
M008	54	F	80	3.0	NSCLC	Absent	Synchronous	Frontal
M257	68	M	90	3.0	NSCLC	Absent	Synchronous	Parietal
M260	67	M	90	3.0	NSCLC	Absent	Synchronous	Occipital
M268	51	M	90	3.5–4.0	NSCLC	Absent	Stable disease	Frontal
M308	56	F	90	3.5–4.0	Breast (HER2+)	Absent	Stable disease	Frontal
M135	27	F	90	3.5–4.0	Breast (HER2−)	Absent	Stable disease	Parietal
M164	53	F	100	1.5–2.5	NSCLC	Present	Synchronous	Frontal

### MRI and analysis

MR imaging was performed on clinical whole body scanners at 1.5T with a single channel head coil. The standard post-contrast T1-weighted sequence was a volumetric fast spoiled gradient echo sequence, which was acquired after the intravenous administration of Gadavist® (Bayer HealthCare, Germany) at a standard dose of 0.1 mmol/kg. The acquisition parameters were as follows: repetition time (TR) 9 ms, echo time (TE) 1.4 ms, flip angle 15 degrees, acquisition matrix 256 × 256, volume 180 × 1 mm axial slices at zero angulation. DWI was obtained using one acquisition over 90 s through single-shot echo planar imaging with b values of 0 and 1000 s/mm^2^. Using the post processing software package GE FuncTool^®^ (v 4.5.5, General Electric Co., Maryland, USA), an ADC map was generated for each dataset. All patients underwent a control volume CT brain on day 1 post operatively as per institutional protocol.

### Stereotactic radiosurgery planning

**Fig. 1:**
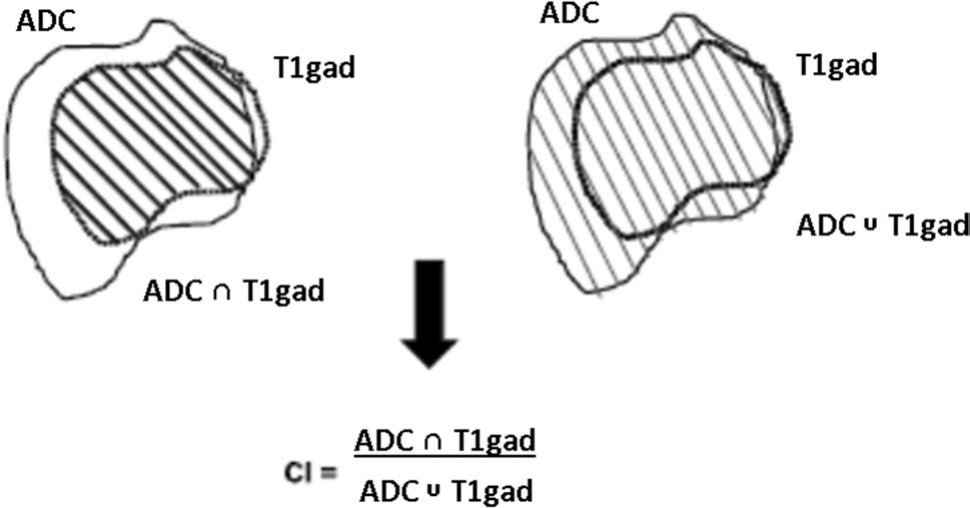
Illustration of the conformity index (=intersection volume ÷ conjunction volume) for comparing treatment volumes based on the T1gad and the ADC studies

Scans were transferred to Oncentra MasterPlan^®^ (Nucletron BV, Veenendaal, Netherlands) and target delineation performed in a randomized reading order by three separate operators experienced in SRS planning and blinded to the clinical information and outcome of the patient. Different imaging sequences were contoured at different days in order to avoid pre-imaging bias. Volumetric analysis was done within MasterPlan^®^ employing the “Case Explorer” tool. We compared treatment volumes based on the post-contrast T1-weighted scans (GTV_T1gad_), using the ADC maps alone (GTV_ADC_) and using both sequences in combination, which we subsequently refer to as diffusion treatment volume (DTV) and then compared these with areas of subsequent recurrence after gross total resection. The conformity index is a concept in radiation oncology used to compare treatment plans and volumes and has a variety of derivations and applications. Here an intentionally simple approach is used, calculating the ratio of intersection of two volumes with their combined total volume to yield an index of their conformity from 0, no similarity whatsoever, to 1, identical plans (illustrated in Fig. 1).

## Results

### Clinical outcomes

All patients studied underwent gross total resection with no complications within 30 days of operation. Median overall survival was 11.8 months (95% CI 7.8–15.8 months) and median time to local recurrence was 8.0 months (95% CI 6.6–9.4 months). In fifteen of seventeen cases neurosurgical resection of a solitary cerebral metastasis was followed by adjuvant whole brain radiotherapy (WBRT) 30 Gy in 10 fractions. In one case the patient was too unwell due to systemic disease to receive WBRT and in one other 20 Gy in 5 fractions was administered at the treating oncologists’ discretion. At recurrence five patients underwent re-operation, three underwent SRS and the remainder received palliative chemotherapy.

### Assessing treatment volumes

Intra-class correlation coefficient showed strong agreement amongst the three different observers; for GTV_T1gad_ alpha = 0.998 (95% CI 0.992–0.999) and for GTV_ADC_ alpha = 0.987 (95% CI 0.958–0.997). Direct pairwise comparison of GTV_T1gad_ and GTV_ADC_ showed no quantitative difference (related samples Wilcoxon signed rank test = −0.45, p = 0.653); values are listed in Table 2. The conformity index (Fig. 1) was used to qualitatively compare the volumes; mean conformity index (±SD) for GTV_T1gad_ compared to GTV_ADC_ was 0.53 (±0.16), which is significantly different from 1 (one sample *t* test, t = −11.9, df = 16, p < 0.001). As a control, the conformity index for GTV_T1gad_ of different observers was calculated and found to be 0.79 (±0.28) with no significant difference from 1 (p > 0.05).

**Table 2:** Average metastasis volume as assessed using the standard post-contrast planning sequence (T1gad) and the ADC map

Case ID	GTV_T1gad_	GTV_ADC_	DTV (union of GTV_T1gad_ and GTV_ADC_)
M011	18.5	29.81	30.46
M013	10.2	12.57	13.65
M019	38.99	27.88	44.82
M028	12.33	9.4	14.23
M031	49.48	42.35	55.36
M033	16.03	10.07	16.89
M042	71.71	64.51	77.21
M052	4.33	4.27	5.77
M222	7.27	6.8	9.31
M302	14.18	18.58	19.87
M008	2.35	1.5	2.37
M257	4.41	5.48	6.70
M260	0.75	0.49	0.84
M268	4.85	7.56	8.18
M308	9.52	7.05	12.45
M135	8.74	15.06	16.35
M164	1.19	1.74	2.24
Median	9.52	9.40	13.65

Volumes are in cm^3^

### Combining the ADC map with standard planning

**Fig. 2:**
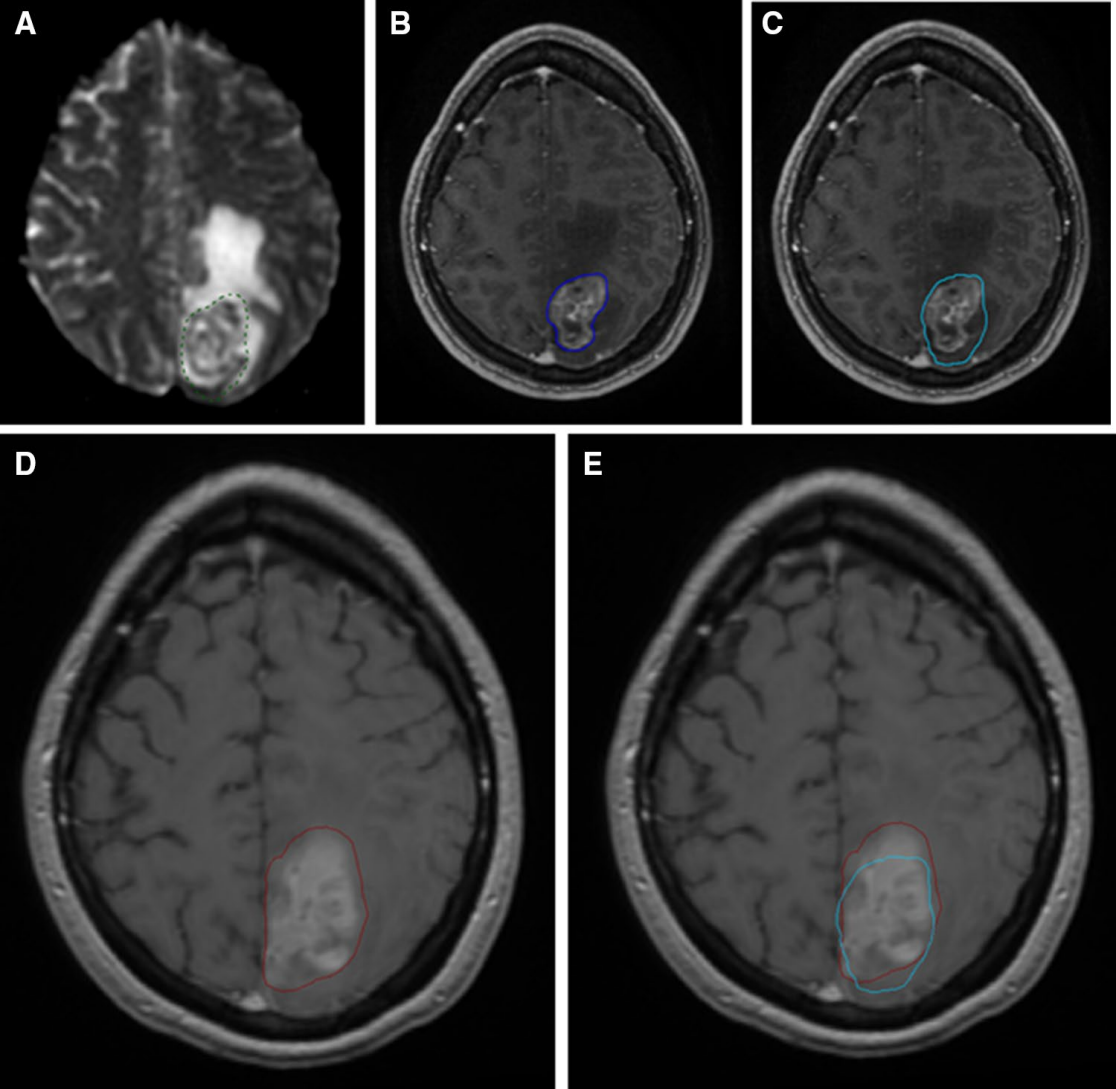
An occipital metastasis in a patient with metastatic melanoma (M-011). Treatment plans were generated based on **a** the ADC map—GTV_ADC_ and **b** the post-contrast T1-weighted planning study—GTV_T1gad_. The combined plan with addition of these two volumes is termed the diffusion treatment volume or DTV (**c**). These volumes are superimposed on post-contrast T1-weighted sequences acquired at the point when the resected tumour recurred. The volume of local recurrence that was covered by the GTV_T1gad_ (**d**) was less than that covered by the DTV (**e**) and differed in conformation

The DTV was significantly greater than the GTV_T1gad_ both quantitatively (related samples Wilcoxon signed rank test = 3.621, p < 0.001) and qualitatively (conformity index = 0.74 (±0.17), significantly different to 1, t = −6.27, p = 0.001). There was no significant difference in the volume of recurrent tumour that would have been covered by the GTV_T1gad_ vs. the GTV_ADC_ (3.53 and 3.57 cm^3^ respectively, Wilcoxon related samples test = −0.052, p = 0.959) and the conformity index was not significantly different (0.20 ± 0.16 for GTV_ADC_ vs. 0.22 ± 0.20 for GTV_T1gad_, paired *t* test = 0.823, p = 0.424). The volume of recurrence encompassed by the DTV was significantly greater than the GTV_T1gad_ in 12 of 16 individual cases as well as overall for the group (median 3.84 cc, range 0–32, vs. 3.53 cc, range 0–31), Wilcoxon related samples test = −3.061, p = 0.002; this is illustrated for one case (M-011) in Fig. 2.

## Discussion

We have shown that using an ADC map in addition to a conventional post-contrast T1-weighted MRI sequence to plan radiosurgery for solitary brain metastases results in a greater treatment volume with a different conformation compared to the standard protocol. Furthermore, this combined treatment volume intersects with a greater volume of subsequent local recurrence of that metastasis following complete resection. This analysis suggests the ADC map to provide additional, different, clinically useful information compared to the post-contrast T1-weighted sequence alone.

Local recurrence occurs in approximately 20% of cases following radiosurgery [17] and poses a clinical problem for patients and clinicians. ADC is known to decrease with increasing cellularity. Unlike arbitrarily increasing the treated volume, which has not consistently been shown to reduce local recurrence [18] and may risk radionecrosis to normal brain [19], using the ADC map to plan SRS may therefore cover biologically active areas, although it is likely that ADC changes represent a generalized overall assessment of the underlying biological and pathological processes. ADC changes have been used in a radiotherapy planning study to distinguish high-risk areas for glioblastoma recurrence [20]; however, to our knowledge DWI has never been applied to metastasis radiosurgery planning before. The only investigations into SRS planning for brain metastases that focussed on combining metabolic with anatomic imaging by using FDG-PET [21, 22]; this study resulted in modified treatment plans for 2/3 of brain metastases in a series of 8 lesions. One further study fitted 11-C methionine PET to the contrast-enhancing border [23] using a simple model to provide metabolic tumour delineation, but did not look at outcome parameters.

There are limitations to our study that need to be taken into account when interpreting the data. Our study design is retrospective in nature, which, however, offered the advantage of a complete follow-up from time of diagnosis to death. Further prospective studies could evaluate SRS planning based on the ADC map and the post-contrast T1-weighted MRI followed by comparison of subsequent local recurrence with the original plans. Nevertheless, our pilot study confirms that diffusion-based plans were of reasonable quality and detail. Moreover, the extent of coverage of such plans in regard to surrounding brain tissue and the technical integration of diffusion-weighted imaging into a planning software could be demonstrated. At this point in time, ADC may not routinely be added for GTV definition in SRS as normal tissue constraints have to be considered and tumour volumes tend to be larger when applying the DTV concept as presented. However, if our preliminary data are confirmed in future studies, this limitation may be overcome by hypo-fractionated stereotactic treatments as the risk–benefit ratio will be improved with increasing tumour size.
